# At discharge gait speed and independence of patients provides a challenges for rehabilitation after total joint arthroplasty: an observational study

**DOI:** 10.1186/s40945-016-0020-6

**Published:** 2016-06-29

**Authors:** Mattia Morri, Emanuela Natali, Daniele Tosarelli

**Affiliations:** Physical Therapy and Rehabilitation Unit, Istituto Ortopedico Rizzoli, Via Pupilli 1, 40136 Bologna, Italy

**Keywords:** Hip replacement arthroplasty, Rehabilitation, Recovery of function, Patient outcome assessment

## Abstract

**Background:**

The level of functioning in people discharged from hospital after hip arthroplasty is very heterogeneous and prognostic factors are not fully understood. The aim of this study was to determine the mean level of autonomy achieved by such patients at discharge from hospital using the Iowa Level of Assistence (ILOA) scale as a measurement tool and to investigate the possible predictive factors of this autonomy.

**Methods:**

It was conducted a prospective cohort study including hip arthroplasty patients treated consecutively in 2012. Hip arthroplasty patients following fractures, revision surgery and partial replacement were excluded, as well as patients with concomitant neurologic or rheumatologic diseases or postoperative complications that did not allow to continue the rehabilitation program, and patients with a hospitalization of more than 7 days. During the last 24 h of hospital stay the physiotherapist filled in the ILOA scale and collected all data (age, gender, number of physiotherapy treatments, length of hospitalization). Statistical analysis (univariate and multivariate analysis) was performed between the variables collected and the ILOA Score.

**Results:**

The sample was composed of 167 patients. The mean score of the ILOA was 16.6 (±6.5) and gait speed had the poorest outcome 0.19 m/s - 0.43 m/s. Multivariate analysis showed that older women are most at risk of not achieving good levels of autonomy.

**Conclusions:**

In hip arthroplasty patients at discharge from hospital gait speed is severely impaired. The challenge for rehabilitation should be to recover walking ability and efficiency starting from the early post-operative period. Gender- and age-tailored rehabilitation programs should be considered by placing particular attention on elderly women.

## Background

Nowadays, the post-operative rehabilitation approach in the treatment of total hip arthroplasty (THA) is applied earlier and is multidisciplinary [1]. Protocols that involve an accelerated and intense physiotherapy intervention enable patients to achieve earlier functional autonomy thus reducing hospitalization time [2–4]. The literature descriptions of the autonomy achieved by patients at discharge from hospital are very heterogeneous [5–7] and often without specific measurement scales and limited to the measurement of single performances such as the ability to walk independently. A complete measurement of the autonomy achieved, however, plays a central role to show precisely the results obtained by early rehabilitation [8]. Several scales measure the autonomy of the patient, as the “Functional Independence Measure” (FIM) [9] and the “Barthel Index” (BI) [10]. These scales, however, include items like feeding, bowels and bladder function, which do not seem relevant for acute patients after joint arthroplasty. The “Iowa Level of Assistance” (ILOA) [11] scale seems more adequate to evaluate the outcome of the early rehabilitation treatment. Assessing 5 functional activities, this scale providesa more complete information about patients’ autonomy. Other commonly used testsget information only about a specific aspect of walking as endurance (e.g., Six-minutes walk test) [12] or gait speed [13–15]. Different authors have used the ILOA scale to measure the early results and to evaluate the efficacy and the optimal intensity of the rehabilitation treatment [16, 17] or the influence of different surgical techniques [18, 19]. The factors that significantly influence the autonomy achieved, however, have not yet been fully explained. Vincent el al. [20], in a retrospective study on 332 THA patients, considered different possible prognostic factors and found that only gender and age were significant. The aim of this study was to determine the mean level of autonomy achieved by patients undergoing total hip arthroplasty at discharge from hospital using the ILOA scale as a measurement tool and to investigate the possible prognostic factors of this autonomy.

## Methods

A prospective study was performed on consecutive primary THA patients following chronic joint disease, between January and May 2012 at department of “Orthopaedic-Traumatology and Prosthetic surgery and revisions of hip and knee implants” of the Rizzoli Orthopaedic Institute, where the postoperative rehabilitation program was implemented. The rehabilitation program for the joint arthroplasty following fractures, revision surgery, and partial replacement differs from the standard, therefore these conditions were excluded from the study. Furthermore, in order to obtain as uniform a sample as possible, patients with concomitant neurologic diseases (Parkinson’s disease or pre-surgery stroke) and rheumatologic diseases (rheumatoid arthritis or ankylosing spondylitis) or postoperative complications that do not allow to continue the rehabilitation program (severe anemia, cardiac problem as acute myocardial infarction, respiratory problems as lung infection) with a hospitalization of more than 7 days were excluded. Institutional review board approval was obtained from the Ethics Committee of the Rizzoli Orthopaedic Institute before conducting this study.

### Sample size

The number of the sample was calculated by the results of a pilot study of 18 cases which gave a mean ILOA score of 20.5 and a standard deviation of 10.46. Accepting a margin of error of 2 points for the Confidence Interval at 95 %, the number of patients needed to be recruited was at least 107.

### Surgical approach

A posterolateral approach with minimal incision (< or = 10 cm) on the trochanteric region, partial detachment of the gluteus medius and section of gluteus minimus was performed. The capsule was opened by a T section. After reduction of the newly inserted prosthetic hip, the posterior capsule and short rotators were separately repaired with use of non-absorbable sutures passed through drill-holes in the greater trochanter.

### Procedure

The postoperative rehabilitation entailed two daily physiotherapy sessions lasting 30 min beginning from the first postoperative day when the drainage tube was removed. The rehabilitation program was early and accelerated with the aim of achieving an upright position on day 1, walking with a frame on day 2 and walking with forearm crutches and, compatibly with the patient’s clinical conditions, ascending 3 steps on day 3. Helping the patient to regain mobility entailed functional rehabilitation exercises in bed and training for autonomy. Passive and active mobilization of the ankle, the knee and the hip joints was provided. Hip mobilization in flexion (until 90°), extension and abduction was performed in different position as supine, prone and on side. Isometric exercises to improve muscles strength, especially of the quadriceps, were conducted at each physiotherapy session on the bed and when possible in standing position. Each physiotherapist was free to choose the activities to carry out in each session within the rehabilitation protocols. During the last 24 h of hospital stay the physiotherapist filled in the appropriate assessment form that includes the ILOA scale and collected the data required for the study: preoperative American Society of Anesthesiologists (ASA) Score for comorbidities, postoperative complications, age, gender, number of physiotherapy treatments, length of hospitalization. The ASA physical status classification system is a system used to assess the fitness of patients before surgery [21]. An ASA score < 3 indicates healthy people or people with mild systemic disease without substantive functional limitations. As clinical practice the physiotherapist performed the ILOA Scale in the ward without knowing the purpose of the study. The other data were collected from the patient’s rehabilitation form and from the anesthesia and medical records.

The primary outcome considered was the level of autonomy achieved by the patients at discharge from hospital, measured by the ILOA scale. This scale takes into account the patient’s performance in 5 functional activity tests: supine-to-sitting, sitting-to-standing, walking, climbing 3 steps, and gait speed. The score of the first four tasks is based on the level of help supplied to the patient by the operator for the safe execution of the activities and it ranged from 0 (independent) to 6 (not tested for safety reasons). The gait speed is rated according to the time taken to cover a distance of 13.4 m with a score from 0 (≤20 s) to 6 (≥70 s). The type of assistive device used in tasks that involve the standing position and ambulation is rated from 0 (no assistance device) to 5 (walking frame). The total score ranges from 0 to 50, where 0 indicates the patient’s complete autonomy and 50 the maximum dependence. The scale was translated into Italian from English, the original language, using the linguistic face validity protocol [22]. The reliability, validity and responsiveness of original version of the ILOA was studied by the authors, who found good intratester (k = .79-.90) and moderate intertester (k = .48-.78) reliability, high correlation with the Harris Hip Rating Scale scores (*r* = -.86) and good responsiveness to 4 day of therapy postoperatively [11]. The ILOA received the best ratings in a systematic review aimed at evaluating the measurement properties of all performance-based tests used to assess the physical function in people with hip or knee osteoarthritis [23]. At discharge the patients were sent to home or other rehabilitation centre, or other structure. The orthopedic doctor responsible of the ward chose the right discharge according to several elements: age and clinical condition, ability to walk and to ascend 3 steps, family situation and patient’s preference. The physiotherapist was not directly involved in this process.

### Statistical analysis

All continuous data are expressed in terms of mean ± SD and categorical variables are expressed as proportions or percentages. The Kolmogorov Smirnov test was performed to test normality of continuous variables. The correlation tests were conducted between the variables collected (age, gender, number of treatments, length of hospitalization) and the ILOA score. Further correlations were studied between the variables and the level of help score for each item of the ILOA scale. One-Way ANOVA was performed to assess the correlation between gender and ILOA Score when the Levene test for homogeneity of variances was not significant (*p* < 0.05), otherwise, the Mann Whitney test (two groups) was performed. The Spearman rank Correlation was used to assess correlation between continuous data (age, number of treatments and length of hospitalization); the Pearson chi square test evaluated by the exact method (to manage small subgroups) was performed to investigate the relationships between grouping variables. The variables that had a significant correlation with the ILOA score, were utilized to perform the multivariate analysis model. The multivariate analysis was performed by the General Linear Model having the fixed effects as the categorical predictor and the covariates as the continuous predictor. For all tests *p* < 0.05 was considered significant. Statistical Analysis was carried out by using the Statistical Package for the Social Sciences (SPSS) software version 15.0 (SPSS Inc., Chicago, USA).

## Results

One hundred eighty six patients were eligible for this study. 19 patients were excluded having a length of stay superior on seven days. The medical records confirmed that they all had postoperative complications: 9 cases for hemorrhagic anemia, 4 cases for cardiac problems, 3 cases for respiratory problems, 1 case for wound infection, 2 cases for other reasons. Among this group the evaluation of the comorbidities identified 53 % of patient with ASA score > 2.

The sample studied was composed of 167 patients, none of which was lost to follow-up. Table 1 shows all the data about the characteristics of the patients and the variables studied. 93 % of patients were admitted with a diagnosis of hip osteoarthritis with pain, reduction of range of motion (ROM), limitation in the daily life activities, especially of walking capacity. The diagnosis of necrosis of the femoral head was for 7 % of cases. The mean ILOA score at discharge was 16.6 (±6.5). The functional items of ascending 3 steps and gait speed were the tests where the patients needed more assistance and had the poorest performance: the mean score of ILOA level of help for these two items was 1.4 (±1.9) and 4.1 (±1.7), respectively. Mean gait speed ranged between 0.19 m/s and 0.43 m/s. At discharge 80 % of patients was sent to home, 13 % to a rehabilitation ward and 7 % to other health structures. The univariate analysis showed a potential association between gender, age and the level of autonomy achieved at discharge (Tables 2 and 3). Conversely, the length of hospitalization and the number of treatments did not show a significant correlation with the ILOA score. A correlation between the number of treatments performed and the supine-to-sitting item was found (Table 3). Age and gender, that had a significant correlation with the ILOA Score, were utilized to perform the multivariate analysis model showed in the Table 4. The two variables were confirmed to be independently associated with the ILOA score. Making an estimate with age set at 60.8 years highlighted an ILOA score of 14.8 (IC 95: 13.3-16.3) for men and 17.7 (IC 95: 16.5-18.9) for women. The ILOA score increased 0.16 points for every year of age.

**Table 1:** Characteristics of the participants. Data are reported as mean (SD), unless otherwise specified

	N (167)
Women, *n* (%)	103 (61.7 %)
Age (year)	60.8 (12.7)
Nr of patients with ASA Score <3, (%)	156 (93.4 %)
Nr. of physioterapy treatments	7.4 (1.6)
Length of hospitalization (day)	5.6 (1.1)
ILOA Score	16.6 (6.6)

**Table 2:** Univariate Analysis: Mann Whitney test

		ILOA	Sup – Sit	Sit – Stand	Walk	3 steps	Speed
		mean ± SD	mean ± SD	mean ± SD	mean ± SD	mean ± SD	mean ± SD
Gender	Men	14.4 ± 4.7	0.25 ± 0.69	0.19 ± 0.47	0.28 ± 0.55	0.97 ± 1.29	3.28 ± 1.89
	Women	18.0 ± 7.1	0.41 ± 0.82	0.33 ± 0.68	0.70 ± 1.22	1.65 ± 2.09	4.63 ± 1.42
	*P* value	<0.0005	n.s.	n.s.	n.s.	n.s.	<0.0005

**Table 3:** Univariate Analysis: Spearman rank Correlation

		ILOA	Sup – Sit	Sit – Stand	Walk	3 steps	Speed
Age	Rho	0.469	0.281	0.289	0.450	0.371	0.445
	*P*-value	<0.0005	<0.0005	<0.0005	<0.0005	<0.0005	<0.0005
Nr treatments	Rho	0.110	0.155	0.018	0.034	0.044	0.097
	*P*-value	n.s.	0.046	n.s.	n.s.	n.s.	n.s.
Length of hospitalization	Rho	0.019	0.019	-0.041	-0.053	-0.015	0.037
	*P*-value	n.s.	n.s.	n.s.	n.s.	n.s.	n.s.

**Table 4:** Multivariate Analysis (General Linear Model) – ILOA Score

	B	Confidence Interval 95 %		Partial eta squared	*P* value
		Inferior limit	Superior limit		
Gender (male)	-2.873	-4.789	-0.956	0.51	0,004
Age	0.16	0.09	0.237	0.105	<0.0005

## Discussion

The aim of the study was to measure the level of autonomy at discharge using the ILOA as a measurement scale and assess any predictive factors. The population enrolled in the present study showed a good level of autonomy. This level was slightly greater than the level reported by Stockton and Mengersen [16], who found a mean score of 18.2 (±7.7) at the ILOA Scale measured on the 6th day. However, the patients assessed by Stokton and Mengersen [16] were slightly older (the mean age was 68.3 years) and with a smaller percentage of women (43.3%).

When studying the postoperative rehabilitation course, Zavadak et al. [8] showed variability in achieving the various functional activities by the patients treated by joint arthroplasty. In particular, ascending and descending 3 steps was found to be the most difficult activity compared to the other activities examined, i.e. walking and bed mobility. The data of the present study showed that supine to sit transfers was an important issue for the patient treated by THA, by revealing a correlation with the number of treatment performed. This can be explained by the surgical technique with the postero-lateral approach which entails the partial detachment of the gluteal musculature and therefore causes an initial difficulty in performing the active abduction movement of the hip. The supine-to-sitting item of the ILOA scale seems particularly sensitive in assessing this aspect. Achieving a normal gait speed is one of the most challenging goals after THA. This ability was not considered by Zavadak et al. [8], but several authors suggested that the measure of the speed of walking is important to evaluate the improvement of patients during different stages of the rehabilitation program [14, 15, 24, 25]. In the present study the gait speed recorded at discharge was strongly impaired. This finding is similar to the results of Lawlor et al. [18]. It has been reported that in the first six months there is a significant improvement of the walking speed, which increases up to 0.82 m/s one month after surgery [13], to 0.95 m/s at six weeks [14] and to 1.14 m/s [14] or 1.08 m/s [15] at six months. Normal gait speed is an evolutionary task that is developed through physiotherapy and exercise and requires multiple abilities, as endurance, muscle strength, balance, coordination, weight bearing control. Other factors as pain, edema, postsurgical weakness and medicines can also affect the performance. Inadequate gait speed causes a poor walking efficiency with a high energy loss [13]. The rehabilitative program should improve the control of the centre of mass of the body in order to smooth its vertical displacement during gait and improve the efficiency of this skill [13]. Early post-surgical rehabilitation protocols usually focus on functional skills as walking independence training, weight bearing restrictions, prevention of implant dislocation and daily life activities. Skills as gait speed, symmetry and cadence are secondary goals that are postponed to the post-acute stage of recovery. In the acute setting of care, because of the short time of hospitalization and patient postsurgical clinical condition, the training of gait speed is a secondary goal that actually is mediated by a personal initiative of the physiotherapist. Specific exercises as respiratory training during walking, behavioural graded activity as endurance training and muscle strength and flexibility during function, pain and disability discrimination, posture change pacing [26] in the early physiotherapy treatment could be useful to increase the gait speed at discharge. Further studies are necessary to verify this hypothesis. In agreement with the literature, the multivariate analysis showed that age and gender were significant factors in determining the functional outcome [20]. Elderly women achieved the poorest performance. Conversely, the length of hospitalization and the number of treatments did not seem to influence directly the autonomy achieved. This finding conflicts with the data published by Ganz et al. [27], who showed how, in the period from 1990 to 2000, with a reduction in hospitalization time there was a reduction in the performance at discharge. New surgical techniques and a more intense rehabilitation approach might have enabled an inversion of this trend. All these indications should be considered to develop more and more specific rehabilitation programs that enable patients to achieve better levels of autonomy at discharge from hospital, bearing in mind that elderly women are the type of patients most at risk in this phase of rehabilitation. Limits of the present study are the enrollment of a selected sample and the lack of data about the preoperative functional ability of the patients. However, according to a recent systematic review [28] the ASA Score is the only factor able to influence the length of stay after total hip arthroplasty. Other preoperative patient related factors do not seem to influence the functional recovery. Since the participants enrolled in the present study had similar ASA scores (93 % had an ASA score <3 points), it is unlikely that their baseline characteristics influenced the results.

## Conclusion

Using a validated measurement scale to assess patients’ functional performance at discharge from hospital gives an indication of the autonomy achieved by the patient which is comparable with other clinical findings and enables to detect the variability of the results obtained. Gait speed is strongly impaired at discharge. The physiotherapy protocol in the acute phase after the surgery should include gait speed as a skill to reach in order to improve walking ability and efficiency. Age and gender were found to be significant prognostic factors of the levels of autonomy at discharge. These findings should be considered in any project aimed at optimizing an accelerated rehabilitation program after THA.
